# *TECRL*: connecting sequence to consequence for a new sudden cardiac death gene

**DOI:** 10.15252/emmm.201606967

**Published:** 2016-10-26

**Authors:** Matthew D Perry, Jamie I Vandenberg

**Affiliations:** ^1^ Mark Cowley Lidwill Research Programme in Cardiac ElectrophysiologyVictor Chang Cardiac Research InstituteDarlinghurstNSWAustralia

**Keywords:** Cardiovascular System, Genetics, Gene Therapy & Genetic Disease

## Abstract

The sudden unexpected death of a child is a devastating event. One of the first questions a family will ask is “Why did this happen?” In some cases, the answer may become obvious during a postmortem examination, but in up to 40% of cases, the postmortem is negative (Bagnall *et al*, 2016). In the last 1–2 decades, an improved understanding of the genetic basis of the primary arrhythmia syndromes, the major cause of sudden unexplained death in children with structurally normal hearts, has greatly enhanced our ability to make a postmortem diagnosis (Van Norstrand & Ackerman, 2010). Establishing an accurate genetic diagnosis can not only answer the parents' question as to why did this happen to my child, but is invaluable for cascade screening of all family members to identify other individuals harbouring the same mutation and who therefore may be at risk of sudden cardiac death. However, even after screening for all of the established genes associated with primary arrhythmia syndromes, up to two thirds of unexplained cardiac deaths will remain unsolved. Such was the case for a family of Sudanese origin with a highly malignant form of exercise‐induced arrhythmias, originally reported by Bhuiyan *et al* (2007).

In recent years, next‐generation sequencing techniques, such as whole‐exome sequencing (WES), have revolutionized our ability to identify genetic variants throughout the genome. However, differentiating between the often many thousands of novel rare variants that are benign coincidental findings and the variants that are the true cause of disease in any given individual can be incredibly challenging (Richards *et al*, 2015). Furthermore, incorrectly associating a genetic variant with a clinical phenotype can be worse than having no diagnosis at all. This can potentially lead to inappropriate treatment of the patient and inaccurate diagnoses in family members or other unrelated individuals harbouring the same variant.

In the case of the Sudanese family reported in this issue of *EMBO Molecular Medicine* (Devalla *et al*, 2016), solving the genetics was made considerably easier by the fact that the condition showed autosomal recessive inheritance and the parents were first cousins. Using whole‐exome sequencing, Devalla and colleagues identified a homozygous loss‐of‐function mutation in a novel gene, called *trans*‐2,3‐enoyl‐CoA reductase‐like (*TECRL*), that was present in all affected family members. Examination of the ExAC database of 60,706 unrelated individuals (Lek *et al*., 2016) shows that heterozygous loss‐of‐function mutations in the *TECRL* gene occur at the expected frequency given the size of the gene. However, there are no reports of homozygous loss‐of‐function mutations in the gene. This is consistent with the parents being heterozygous carriers of the loss‐of‐function mutations not having a phenotype whilst the children with a homozygous loss of *TECRL* function have severe disease.

Devalla *et al* (2016) also identified two other unrelated patients, both of whom had experienced stress‐ or exercise‐induced arrhythmias with aborted sudden cardiac arrest, who were also found to be carriers of a novel homozygous mutation in the *TECRL* gene. In all three case studies, the clinical phenotypes showed overlapping characteristics of two primary arrhythmia syndromes: long QT syndrome (LQTS) and catecholaminergic polymorphic ventricular tachycardia (CPVT). This highlights the difficulty in making clinical diagnosis in the absence of an established genetic association. However, finding a genetic association, even one as strong as it is in this Sudanese family, is just the first step in establishing whether this is indeed the cause of the disease. To address this more difficult question, Devalla and colleagues generated human induced pluripotent stem cells (hiPSCs) from both affected and unaffected individuals in the family and used these lines to derive cardiac myocytes for functional studies (Devalla *et al*, 2016). Human iPSC‐derived cardiac myocytes provide an ideal platform for establishing causally cohesive links between genotypes and cellular phenotypes as they contain all of the signalling and electromechanical properties of human cardiac myocytes.

Analysis of the homozygous *TECRL* mutation in iPSCs revealed altered properties, including an elevated diastolic Ca^2+^, smaller amplitude and slower decay of cytosolic Ca^2+^ transients, and a prolonged action potential duration (APD). Noradrenaline also increased the propensity for triggered arrhythmia caused by delayed after depolarizations in the cardiac myocytes derived from the homozygous iPSC lines. The authors also showed that knock‐down of *TECRL* using shRNA in wild‐type embryonic stem cell‐derived cardiac myocytes produced a similar phenotype. Another distinct advantage of using iPSC‐derived cardiac myocytes to establish genotype–phenotype relationships is that they provide a platform with which to test pharmaceutical therapies. Previous work has established that class I antiarrhythmic drugs are potentially useful in the treatment of CPVT (Watanabe *et al*, 2009), and in this study, Devalla and colleagues found that flecainide was able to reverse the phenotype in the cardiac myocytes derived from the homozygous *TECRL* loss‐of‐function iPSC lines.

The authors have provided compelling data linking homozygous loss‐of‐function mutations in *TECRL* to the pro‐arrhythmia phenotype in this Sudanese family. Although they did not generate iPS cell‐derived cardiomyocytes from the Canadian individuals with *TECRL* mutations, it is reasonable to assume that they would very likely have the same phenotype. However, the big unknown is what is the precise function of TECRL. The authors provide evidence to show that it is located in the heart and preferentially expressed in the endoplasmic reticulum. Given its homology to proteins involved in lipid metabolism, it is possible that TECRL may also be involved in lipid metabolism, but this needs to be confirmed. Whilst most primary arrhythmia syndromes are associated with mutations in ion channels or calcium handling proteins, there is precedence for mutations in metabolic genes being associated with cardiac arrhythmias (Ahmad *et al*, 2005). Other examples include mutations in PRKAG2 (AMP‐activated protein kinase) associated with Wolff–Parkinson–White syndrome (Gollob *et al*, 2001), and glycogen storage diseases such as Pompe disease (a recessive lysosomal acid α‐1,4‐glucosidase deficiency), Fabry disease (X‐linked lysosomal hydrolase α‐galactosidase A deficiency) and Danon disease (X‐linked lysosome‐associated membrane protein 2 (LAMP2) deficiency) (Ahmad *et al*, 2005). Understanding the precise function of TECRL both in health and disease and how defects in TECRL result in altered levels of calcium handling proteins and hence altered calcium homoeostasis should be a fruitful avenue for future research with the possibility of providing avenues for the development of new therapeutics.

The authors have now solved the riddle for the cause of the sudden deaths in this family. But what are the implications beyond the family? First, they have identified a new inherited arrhythmia syndrome gene, *TECRL*, that can be added to the panel of genes for routine clinical testing. Second, whilst homozygous mutations in *TECRL* are likely to be extremely rare (on the order of 1 per million, based on frequency of LOF alleles in the ExAC database), it is possible that the 0.1% of individuals with heterozygous loss‐of‐function mutations could have a subtle phenotype that impacts the manifestation of other arrhythmia syndromes.

The authors of this study are to be commended on their comprehensive detective work that has not only benefited the family concerned, but also it has led to the discovery of a new sudden death gene and identified a potential link between lipid metabolism and calcium homoeostasis, which should open up a new avenue for research. Lastly, this study provides further support for the concept of using patient‐specific iPSC‐derived cardiac myocytes to test personalized pharmacological therapies (Collins & Varmus, 2015).
